# Deciphering the Impact of Mutations on the Binding Efficacy of SARS-CoV-2 Omicron and Delta Variants With Human ACE2 Receptor

**DOI:** 10.3389/fchem.2022.892093

**Published:** 2022-06-08

**Authors:** Alamgir Khan, Salman Ali Khan, Komal Zia, Mezna Saleh Altowyan, Assem Barakat, Zaheer Ul-Haq

**Affiliations:** ^1^ H.E.J. Research Institute of Chemistry, International Center for Chemical and Biological Sciences, University of Karachi, Karachi, Pakistan; ^2^ Dr. Panjwani Center for Molecular Medicine and Drug Research, International Center for Chemical and Biological Sciences, University of Karachi, Karachi, Pakistan; ^3^ Department of Chemistry, College of Science, Princess Nourah bint Abdulrahman University, Riyadh, Saudi Arabia; ^4^ Department of Chemistry, College of Science, King Saud University, Riyadh, Saudi Arabia; ^5^ Department of Chemistry, Faculty of Science, Alexandria University, Alexandria, Egypt

**Keywords:** SARS-CoV-2, Delta, Omicron, variants of concern, MD simulation

## Abstract

The pandemic of COVID-19, caused by SARS-CoV-2, has globally affected the human health and economy. Since the emergence of the novel coronavirus SARS-CoV-2, the life-threatening virus continues to mutate and evolve. Irrespective of acquired natural immunity and vaccine-induced immunity, the emerging multiple variants are growing exponentially, crossing the territorial barriers of the modern world. The rapid emergence of SARS-CoV-2 multiple variants challenges global researchers regarding the efficacy of available vaccines and variant transmissibility. SARS-CoV-2 surface-anchored S-protein recognizes and interacts with the host-cell ACE2, facilitating viral adherence and entrance into the cell. Understanding the interfacial interactions between the spike protein of SARS-CoV-2 variants and human ACE2 receptor is important for the design and development of antiviral therapeutics against SARS-CoV-2 emerging variants. Despite extensive research, the crucial determinants related to the molecular interactions between the spike protein of SARS-CoV-2 variants and host receptors are poorly understood. Thus, in this study, we explore the comparative interfacial binding pattern of SARS-CoV-2 spike RBD of wild type, Delta, and Omicron with the human ACE2 receptor to determine the crucial determinants at the atomistic level, using MD simulation and MM/GBSA energy calculations. Based on our findings, the substitution of Q493R, G496S, Q498R, and Y505H induced internal conformational changes in Omicron spike RBD, which leads to higher binding affinity than Delta spike RBD with the human ACE2 receptor, eventually contributing to higher transmission and infectivity. Taken together, these results could be used for the structure-based design of effective antiviral therapeutics against SARS-CoV-2 variants.

## Introduction

The COVID-19 global pandemic caused by severe acute respiratory syndrome virus type 2 (SARS-CoV-2) had a catastrophic impact on the global health and socioeconomic system (Acter et al., 2020; Raoult et al., 2020; Sironi et al., 2020). Recently, a new SARS-CoV-2 “heavily mutated” variant was identified in Botswana and South Africa on 11 November 2021. The WHO Technical Advisory Group on SARS-CoV-2 virus evolution classified the new variant lineage B.1.1.529 (PANGO) as “variants of concern” and named as the Omicron variant (Callaway, 2021; Thakur and Kanta Ratho, 2021; Poudel et al., 2022).

**FIGURE 1:**
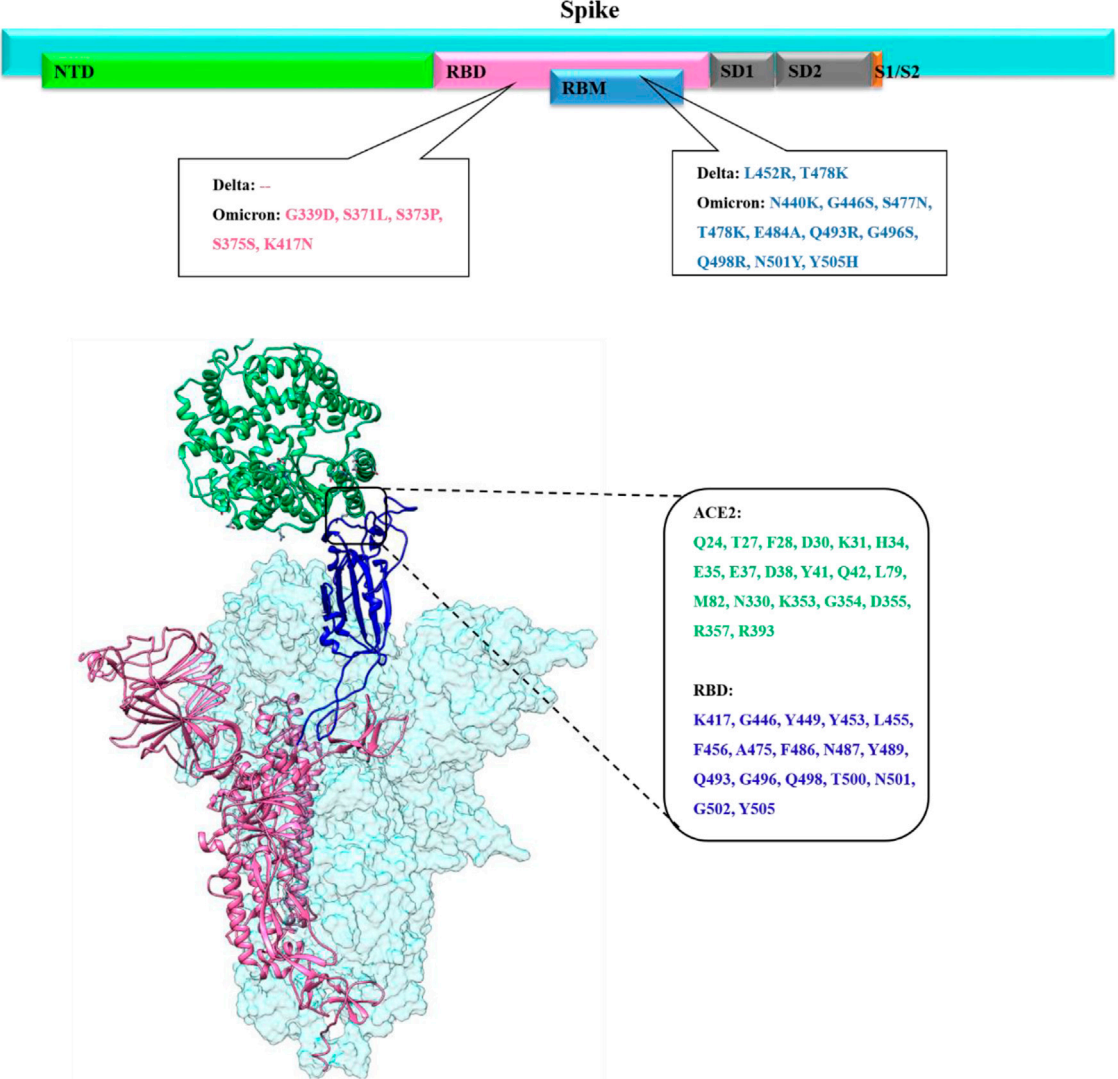
Upper panel shows the overall primary structure of the spike protein of SARS-CoV-2 shown in cyan color while each domain is shown in different colors (N-Terminal domain (NED) in light green, receptor binding domain (RBD) in pink, receptor binding motif (RBM) in blue, subdomain 1 and subdomain 2 in gray, subunit1/subunit2 site in orange color with impactful mutations in Delta and Omicron. The lower panel presents the binding mode and interfacial residues between RBD (up conformation) of the spike monomer with the host ACE2 receptor.

SARS-CoV-2 single-stranded RNA viral genome encodes 16 non-structural proteins (16NSP) and four structural proteins: nucleocapsid (N), membrane (M), envelope (E), and spike (S) proteins (Brant et al., 2021; Zia et al., 2021). The spike glycoprotein is a trimeric protein, and its each monomer is subdivided into S1 and S2 subunits. The S1 subunit contains the N-terminal domain (NTD) and the receptor-binding domain (RBD), and both play key roles in viral attachment, fusion, entry, and transmission (Figure 1) (Santopolo et al., 2021). Particularly, RBD is involved in the recognition of host-cell surface receptor angiotensin-converting enzyme 2 (ACE2). The S2 subunit includes the fusion machinery which undergoes broad conformational changes to drive virus–host membrane fusion, allowing genome delivery and initiation of infection in the host cell (Huang et al., 2020; Walls et al., 2020). Thus, spike protein, specifically the S1 subunit, is a primary target for vaccine development and antibody-based therapeutics against all the SARS-CoV-2 variants (Premkumar et al., 2020).

Several SARS-CoV-2 variants emerged during the COVID-19 pandemic, and the SARS-CoV-2 genome continues to mutate and evolve despite having genetic proofreading mechanisms that keep their genomes intact (Peacock et al., 2021). SARS-CoV-2 global epidemiological genetic surveillance data revealed that the emergence of immunogenically distinct variants was linked to its multiply mutated RNA genome (Islam et al., 2020). These genetic variations within the spike protein, specifically in the RBD domain, can change the conformational equilibrium of the spike-RBD/ACE2 complex which in turn affects SARS-CoV-2 infectivity (Khan A. et al., 2021). The SARS-CoV-2 spike trimeric protein is highly flexible, flipping between close/down and open/up conformation. Spike RBD must be in an upward state to be exposed to ACE2 and only up conformation mediates the favorable binding of the spike protein with the host ACE2 receptor (Figure 1) (Wang et al., 2021; Xu et al., 2021).

All previous SARS-CoV-2 variants of concern (VOCs) including, B.1.1.7 (alpha) in the United Kingdom, B.1.351 (beta) in South Africa, P.1 (gamma) in Brazil, B.1.617 (Delta) in India, and Omicron in Botswana emerge at the time when global population acquired natural immunity and vaccine-induced immunity. The main concerns about the highly mutated Omicron variant emergence in the vaccine-induced immunization phase is whether it is more contagious or severe than other VOCs, or whether it can evade vaccine protection. Before Omicron variant emergence, the Delta was the dominant SARS-CoV-2 variant harboring a total of nine mutations (T19R, E156G, Δ157–158, L452R, T478K, D614G, P681R, and D950N) in the spike protein, among which two are in RBD (L452R and T478K) (Figure 1) (McCallum et al., 2021; Teyssou et al., 2021; Wilhelm et al., 2021). The substitution of leucine to arginine at position 452 in spike-RBD, confers a stronger affinity of the spike toward the host ACE2 receptor and decreases immune system recognition capability.

Omicron’s spike protein mutational profile characterization indicate 30 amino acid mutations, along with several substitution three small deletions and one small insertion (A67V, Δ69–70, T95I, G142D, Δ143-145, Δ211, L212I, ins214EPE, G339D, S371L, S373P, S375F, K417N, N440K, G446S, S477N, T478K, E484A, Q493K, G496S, Q498R, N501Y, Y505H, T547K, D614G, H655Y, N679K, P681H, N764K, D796Y, N856K, Q954H, N969K, and L981F) (Karim and Karim, 2021; Lupala et al., 2021). Out of these, 15 of which are in RBD, a critical region governing both binding with ACE2 and antibody binding (Figure 1).

The comparative sequence analysis of Omicron shows that some mutations such as 69–70del, T95I, G142D/143–145del, K417N, T478K, N501Y, N655Y, N679K, and P681H overlap with the mutation of alpha, beta, gamma, and Delta VOCs (Karim and Karim, 2021). These deletions and substitutions are known to increase contagiousness, higher viral binding affinity, and higher antibody escape ability. However, the clear knowledge of crucial estimates of mutation on the binding affinity to the host and vaccine efficacy is still unknown. In this study, we evaluate the impact of mutations on the binding efficacy of the spike protein of SARS-CoV-2 wild type (WT), Delta, and Omicron variants to the human ACE2 receptor using MD simulation and binding free energy calculations to facilitate effective therapeutic development against VOCs.

## Materials and Methods

### Structural Retrieval and Complex Preparation

The SARS-COV-2 genomic sequence of Omicron and Delta variants was retrieved from global initiative on sharing all influenza data (GISAID) under the accession no. EPI ISL 8240498 and EPI ISL 7542798 reported by the Department of Virology, National Institute of Health, Islamabad, Pakistan, respectively (Khare et al., 2021). The EMBL-EBI Transeq (EMBOSS) (https://www.ebi.ac.uk/Tools/emboss/) tool was used to translate the Delta and Omicron spike gene nucleotide sequence into the amino acid sequence. Furthermore, the sequences of both the variants were aligned to the Wuhan wild-type spike protein sequence retrieved from NCBI and performed using the Clustal Omega tool to verify the mutational profile of both the mutants. Furthermore, the sequences of both variants were subjected to the iterative threading assembly refinement server (i-TASSER) (https://zhanggroup.org/I-TASSER/) for 3D model generation (Yang and Zhang, 2015; Khan et al., 2021). The generated models were optimized by energy minimization using molecular mechanics force fields. For WT, the 3D structure was retrieved from the CHARMM-GUI COVID-19 repository (PDB ID 6VSB) (Wrapp et al., 2020). For RBD-ACE2 complex preparation, PDB ID 6M0J was utilized (Lan et al., 2020). The RBD of WT, Delta, and Omicron was superimposed to RBD-ACE2 of 6M0J. Furthermore, the complexes were prepared using the protein preparation wizard of Molecular Operating Environment (MOE v2019.01). This preparation involves assigning the correct bond order, unwanted water molecule removal, terminal capping, adding missing atoms, energy minimization, and assigning partial charges using the AMBER99 force field. Missing hydrogen atoms were also incorporated at the standard protonation state pH 7.

### Molecular Dynamics Simulation

The molecular dynamics study of wild-type SARS-COV-2 strain and mutants such as Omicron and Delta strains was carried out by AMBER. The structural dynamics of mutants and wild-type strains was evaluated using the solvated system neutralized by counter ions. For the achievements of all atom molecular dynamics simulation FF19SB force field and energy refinement, AMBER20 simulation package was used (Salomon-Ferrer et al., 2013). The energy minimization step was being used to address the bad clashes in the systems using steepest descent and conjugate gradient algorithms. At 1 atmospheric pressure with weak restrain and 300 K temperature, all systems were equilibrated. The final production run was executed for 100ns each in the PME (particle-mesh Ewald) algorithm and the cutoff distance of 10Å was used, to treat the long range electrostatic interactions. A graphical processing unit (GPU) accelerated simulation was performed and MD trajectories were subjected to post-simulation analysis using CPPTRAJ and PTRAJ packages.

### Binding Free Energy Calculation

The binding free energy is often used to estimate the affinities of various macromolecular complexes, such as protein–protein and protein–ligand interactions. To explore the binding affinities of SARS-CoV-2 WT, Delta, and Omicron variants, we employed the molecular mechanics generalized Born surface area (MM/GBSA) method by extracting the 1,000 snapshots from the last 10 ns of simulation trajectories (Wang et al., 2019). MM-GBSA. py script, electrostatic, van der Waals (vdW), solvation energy, and total free energy were estimated using the following equation.

ΔG(bind)=ΔG(complex)−[ΔGreceptor+ΔG(ligand)]

G=G(bonded)+G(electrostatic)+G(vanderWaals)+G(polar)+G(nonpolar).

## Results and Discussion

Since the outbreak of COVID-19, SARS-CoV-2 continues to mutate and evolve leading to the emergence of multiple VOCs including alpha, beta, gamma, Delta, and more recently Omicron. These VOCs have shown an essential impact on the infectivity and pathogenicity of the life-threatening virus (Mohandas et al., 2021; Rotondo et al., 2021; Yadav et al., 2021; Stone et al., 2022). The clear knowledge of the crucial estimates of mutation on the binding affinity to the host and vaccine efficacy is still unknown. In this study, we evaluate the impact of mutations on the binding efficacy of the spike protein of SARS-CoV-2 WT, Delta, and Omicron variants with the host ACE2 receptor using MD simulation and binding free energy calculations to facilitate effective therapeutic development against VOCs.

### Molecular Dynamics Simulation

#### Structural Stability of the RBD-ACE2 Complex

**FIGURE 2:**
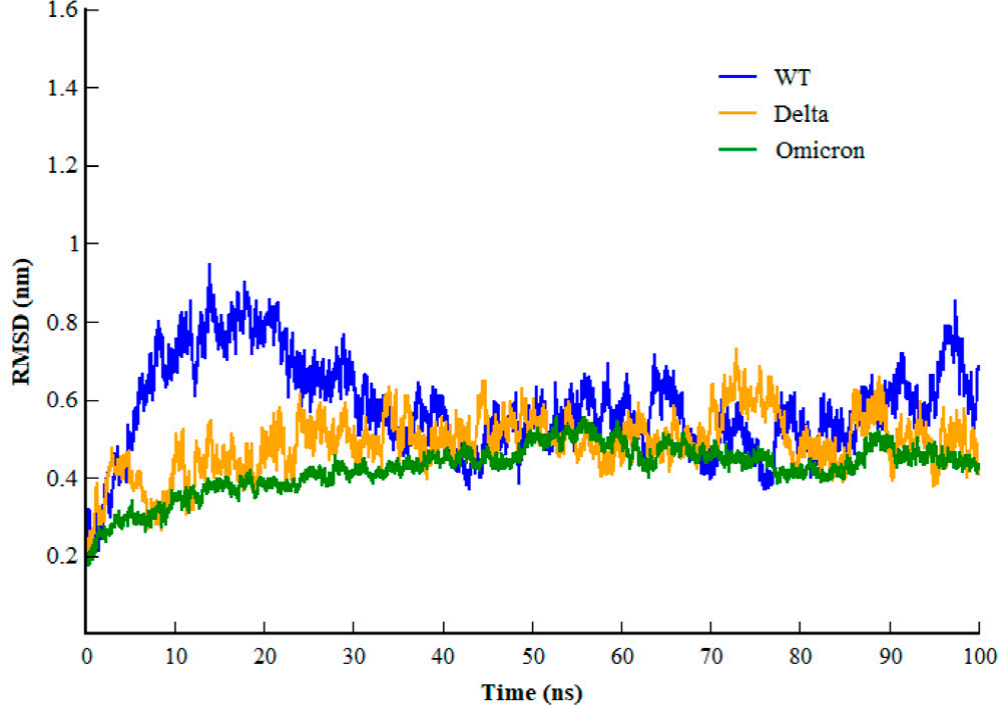
Root mean square deviation of backbone carbon atom of SARS-CoV-2 WT, Delta, and Omicron variants during the course of 100 ns of simulation.

**FIGURE 3:**
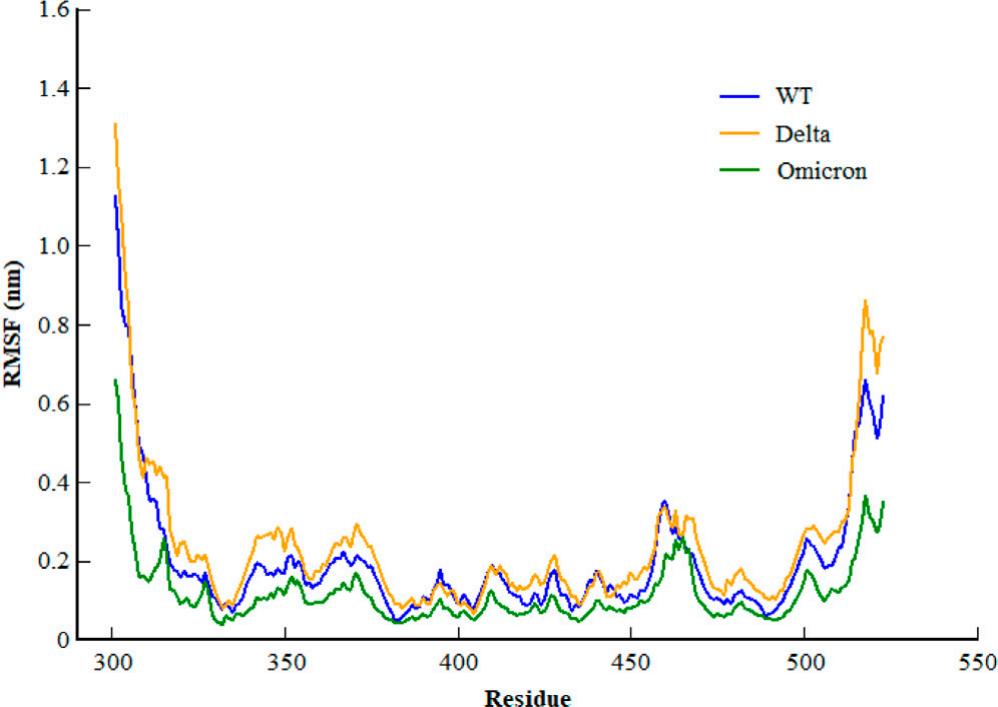
Root mean square fluctuation of backbone carbon atom of SARS-CoV-2 WT, Delta, and Omicron variants during the course of 100ns of simulation.

**FIGURE 4:**
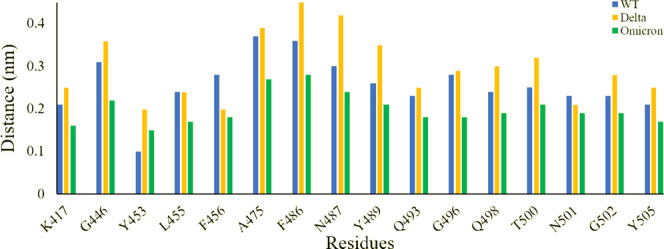
Root mean square fluctuation of receptor binding motifs of SARS-CoV-2 WT, Delta, and Omicron variants.

Inspecting the dynamics and complex stability of the spike protein and host ACE2 receptor, RMSD was evaluated for the simulated trajectories of 100ns. As evident from Figure 2, all the three systems showed variable fluctuations until the end of the simulation with the RMSD of less than 1 nm. WT projected the average RMSD of 0.55 ± 0.07 nm while Delta and Omicron variants projected the average RMSD of 0.51 ± 0.06nm and 0.46 ± 0.03nm, respectively. Initially, WT showed increased RMSD; however, after 20ns, the RMSD gradually decreased and showed variable fluctuations until the end of the simulation. Similarly, the Delta also showed variable fluctuations throughout the simulation. Comparatively, Omicron showed stable RMSD with inconsiderable fluctuations during the whole simulation. Furthermore, to evaluate intrinsic and mutation-induced flexibility of RBD domain residues, RMSF was calculated. The plot showed a similar pattern of fluctuations with different magnitudes for all the three simulated systems (Figure 3). WT projected the average RMSF of 0.24 ± 0.02 nm while Delta and Omicron variants projected the average RMSF of 0.29 ± 0.23nm and 0.14 ± 0.01nm, respectively. In addition to terminal loops, all the systems showed higher fluctuations in two regions; N334-Y380 and L455-T470, which mostly consist of loops. Figure 4 closely shows the mutation-induced flexibility of receptor binding motifs of WT, Delta, and Omicron. Comparatively, interfacial RBD residues of WT, Delta, and Omicron showed similar RMSF. However, the magnitude of fluctuations for Omicron residues was lesser than that of the WT and Delta. These observations were consistent with the previous findings (Lupala et al., 2021; Ortega et al., 2021).

### Interfacial Binding Interactions Between RBD and the Host ACE2 Receptor

**FIGURE 5:**
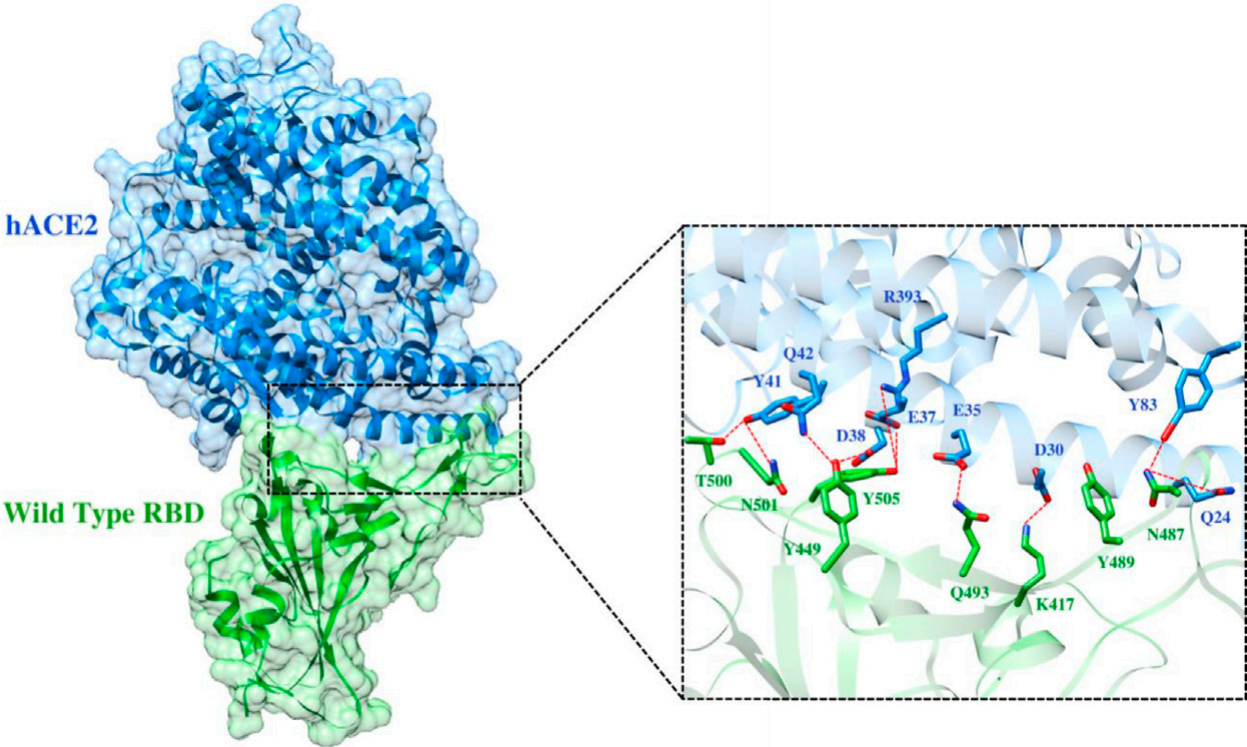
Interfacial binding interactions between WT RBD and ACE2 receptor. RBD and ACE2 residues are colored as green and blue sticks, respectively while red dotted lines represent the hydrogen bonds.

**FIGURE 6:**
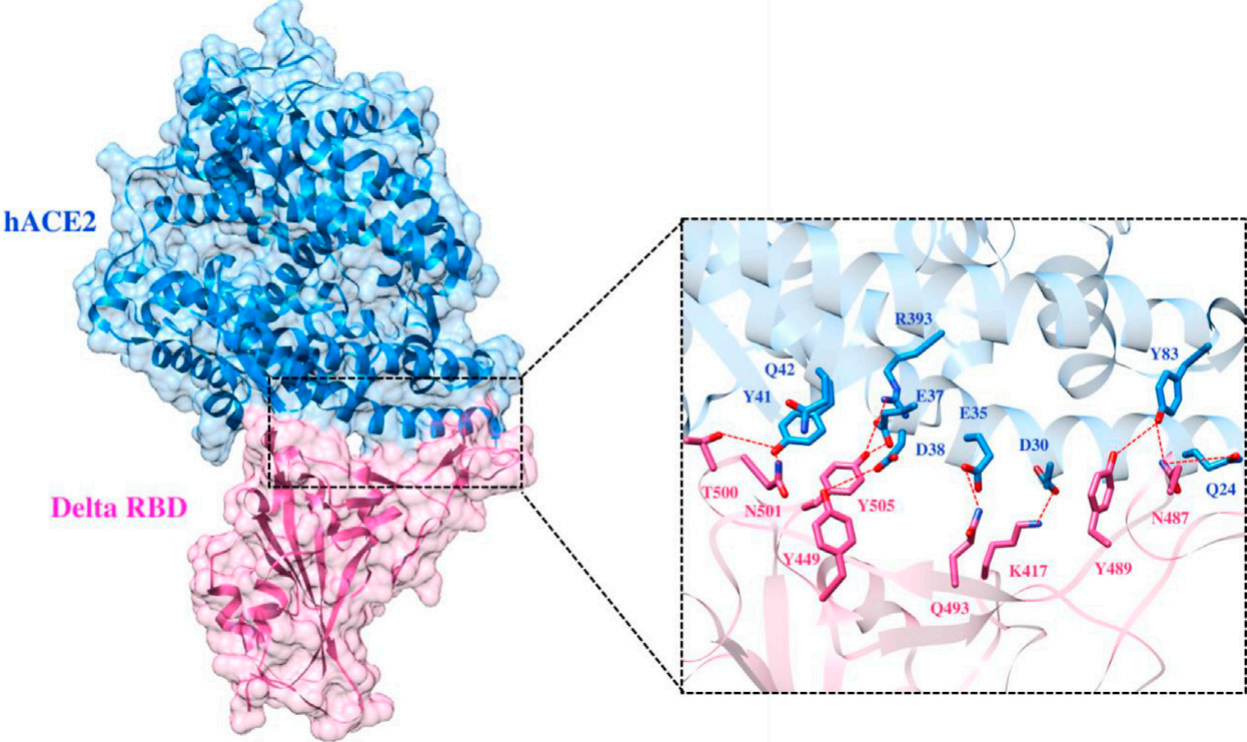
Interfacial binding interactions between Delta RBD and ACE2 receptor. RBD and ACE2 residues are colored as magenta and blue sticks, respectively, while red dotted lines represent the hydrogen bonds.

**FIGURE 7:**
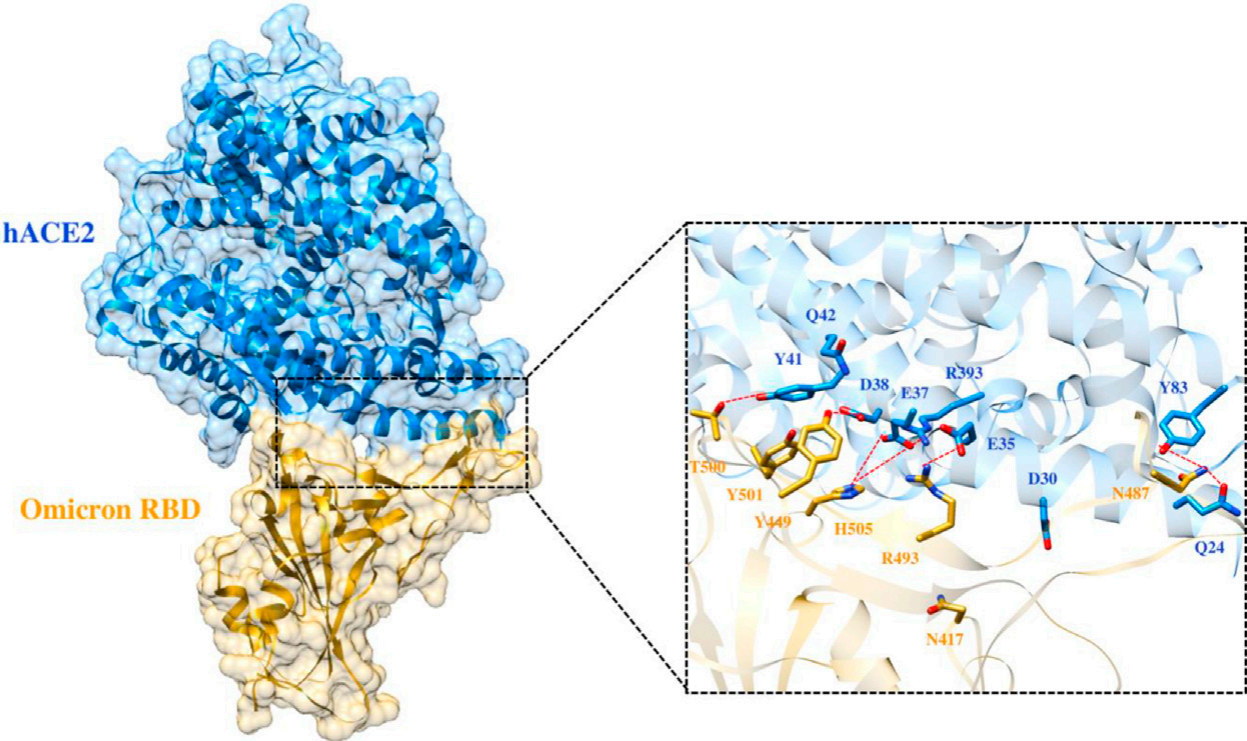
Interfacial binding interactions between Omicron RBD and ACE2 receptor. RBD and ACE2 residues are colored as gold and blue sticks, respectively, while red dotted lines represent the hydrogen bonds.

To elucidate the impact of mutations on the interfacial binding interactions between RBD of SARS-CoV-2 WT and variants with the host ACE2 receptor, the simulated trajectories were analyzed with the cutoff distance of 4 Å. Table 1 and Figures 5–7 depicted the intermolecular interactions between RBD of WT, Delta, and Omicron with the ACE2 receptor, respectively. Taking insight into the intermolecular interactions reveals that RBD-ACE2 complexes of all the three simulated systems were mainly stabilized by a number of significant hydrogen bonds. It has been reported that K417 mutation to N417 and G446 mutation to S446 in the RBD resulted in the loss of the salt bridge with D30 and hydrogen bond with Q42 of hACE2, respectively, and decreased hACE2 binding affinity (Han et al., 2021; Han et al., 2022). Consistent with the previous findings, it was observed that K417 of WT and Delta variants form a salt bridge with D30 of ACE2 with significant occupancy, while in the case of Omicron, K417N did not form any salt bridge throughout the course of simulation. Similarly, G446 of WT consistently mediates a hydrogen bond with Q42 of the ACE2 receptor during the whole simulation while in the case of both the variants no interaction was observed between G446 (G446S in Omicron) and Q42 during the simulation. Y449 of WT and Omicron establishes hydrogen bond contacts with D38 and Q42 of host ACE2 with significant occupancy. In contrast to previous findings, Y499 of Delta variant mediates a hydrogen bond with D38 while no interaction was observed with Q42 (Lan et al., 2020). Moreover, N487 and Y489 of all the three systems interact with Y83 of the host ACE2 receptor by mediating the hydrogen bond contact. Q493 of WT and Q493R of Omicron interact with E35 of the ACE2 receptor with significant occupancy while the bond between Q493 of Delta and E35 of ACE2 was observed with low occupancy during the simulation time. T500 of all the three systems mediates a hydrogen bond with Y41 of the ACE2 receptor with low occupancy. Similarly, N501 of WT and Delta mediates a consistent hydrogen bond with Y41 of ACE2. However, N501Y of Omicron mediates *π*-π with Y41 of ACE2. It was observed that G502 and Y505 of WT, Delta, and Omicron establish hydrogen bonds with K353 and R393 of the ACE2 receptor with significant occupancy, respectively. It has been documented that position altering caused by Y505H in the RBD of Omicron deprives the Y505 hydrogen bond (Liu et al., 2021; Han et al., 2022). In our study, it was observed that Y505H mutation in Omicron plays a critical role in stabilizing the complex by mediating the intermolecular hydrogen bond with 98% occupancy. In addition, Y442, L472, N479, T487, L455, F486, and Q498 of RBD were observed to mediate hydrophobic interactions. Our finding was consistent with previous studies (Antony and Vijayan, 2021; Khan M. I. et al., 2021; Celik et al., 2021; Kumar et al., 2021). However, some contradicting observations were also found. In summary, key substitutions; Q493R and Y505H, that are not previously highlighted help the Omicron to bind more tightly with the ACE2 receptor and weaken the antibody binding to escape the immune response. These findings pave the way for the effective design of antiviral therapy against SARS-CoV-2 variants.

**TABLE 1:** Comprehensive detail of intermolecular interaction of interfacial residues of RDB of WT, Delta, and Omicron with human ACE2 receptor.

Complex	RDB residue	ACE2 residue	Bond type	Distance (Å)	Occupancy (%)
**WT RDB-ACE2**	K417	D30	Salt bridge	3.05	85
	G446	Q42	Hydrogen bond	2.92	79
	Y449	D38	Hydrogen bond	1.75	80
	Y449	Q42	Hydrogen bond	3.57	85
	N487	Q24	Hydrogen bond	3.13	90
	N487	Y83	Hydrogen bond	2.18	94
	Y489	Y83	-	-	-
	Q493	E35	Hydrogen bond	2.53	51
	T500	Y41	Hydrogen bond	2.57	59
	N501	Y41	Hydrogen bond	3.24	85
	G502	K353	Hydrogen bond	1.99	78
	Y505	E37	Hydrogen bond	1.75	50
	Y505	R393	Hydrogen bond	3.02	73
**Delta RDB-ACE2**	K417	D30	Salt bridge	3.22	91
	G446	Q42	-	-	-
	Y449	D38	Hydrogen bond	2.84	88
	Y449	Q42	-	-	-
	N487	Q24	Hydrogen bond	3.61	68
	N487	Y83	Hydrogen bond	3.27	70
	Y489	Y83	Hydrogen bond	3.70	65
	Q493	E35	Hydrogen bond	2.96	44
	T500	Y41	Hydrogen bond	3.56	62
	N501	Y41	Hydrogen bond	1.94	89
	G502	K353	Hydrogen bond	3.22	85
	Y505	E37	Hydrogen bond	2.12	81
	Y505	R393	Hydrogen bond	3.11	82
**Omicron RBD-ACE2**	N417	D30	-	-	-
	S446	Q42	-	-	-
	Y449	D38	Hydrogen bond	1.93	70
	Y449	Q42	Hydrogen bond	2.92	77
	N487	Q24	Hydrogen bond	2.34	82
	N487	Y83	Hydrogen bond	2.76	80
	Y489	Y83	Hydrogen bond	2.53	75
	R493	E35	Hydrogen bond	2.66	83
	T500	Y41	Hydrogen bond	2.25	62
	Y501	Y41	π-π	3.52	85
	G502	K353	Hydrogen bond	2.97	76
	H505	E37	Hydrogen bond	1.82	95
	H505	R393	Hydrogen bond	3.22	98

### Binding Free Energy

To evaluate the impact of mutations of the spike protein on the binding affinity with the host ACE2 receptor, we calculated the binding free energy of SARS-CoV-2 WT, Delta, and Omicron with the ACE2 receptor. It was interesting to note that the heavily mutated Omicron variant showed the highest binding affinity of -52.50 kcal/mol with the ACE2 receptor. However, this magnitude was decreased to -29.21 kcal/mol and -26.62 kcal/mol for the Delta variant and WT, respectively. In order to understand the contribution of individual energy terms in the binding, total binding free energy was decomposed into the individual energy component. The results indicated that the major contributors were electrostatic and van der Waals interactions while the polar component of solvation contributed unfavorably to the binding of all three complexes (Table 2).

**TABLE 2:** Contribution of individual energy component in the binding of SARS-CoV-2 WT, Delta, and Omicron with the human ACE2 receptor.

Complex	vdW kcal/mol	Electrostatic kcal/mol	EGB kcal/mol	Esurf kcal/mol	ΔG solvated kcal/mol	ΔG binding kcal/mol
**WT RBD-ACE2**	-79.99	-1,330.09	1,394.96	-15.38	1,379.58	-26.62
**Delta RBD-ACE2**	-85.60	-1,669.66	1738.70	-12.65	1726.05	-29.21
**Omicron RBD-ACE2**	-102.59	-1980.67	2044.90	-14.13	2030.76	-52.50

**FIGURE 8:**
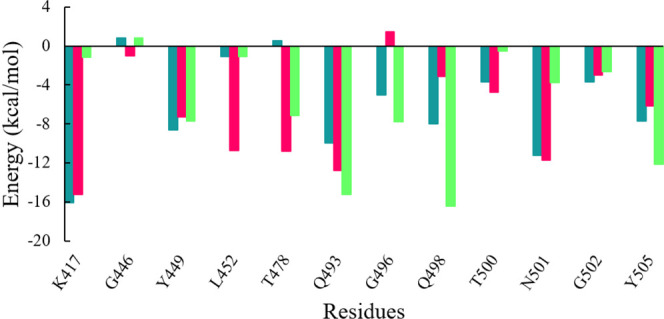
Energy contribution of interfacial residues of RBD of WT (sea green), Delta (violet red), and Omicron variants (lime green).

Further to evaluate crucial residues involved in the strong binding of RBD with ACE2, the total binding free energies were decomposed into the individual residue energy contribution. The energy contribution of interfacial residues of RBD of three simulated systems involved in the binding with ACE2 receptors is shown in Figure 8. In the case of WT, it was observed that K417 most significantly contributed to the binding energy of the WT RBD-ACE2 complex with the value of -16.11 kcal/mol followed by the Delta variant with the value of -15.26 kcal/mol by mediating a salt bridge. However, K417N contributed to the binding of the Omicron RBD-ACE2 complex with the value of -1.18 kcal/mol, which is significantly smaller than the others. G446 contributed nothing or very less to the binding energy of WT, Delta, and Omicron RBD-ACE2 complexes with the value of 0.83 kcal/mol, -1.02 kcal/mol, and 0.79 kcal/mol, respectively. On the contrary, Y449 was energetically stable for WT, Delta, and Omicron with the value of -8.60 kcal/mol, -7.29 kcal/mol, and -7.72 kcal/mol, respectively. L452 and T487 which are mutated to L452R in Delta and T487K in both variants showed the highest energy for the Delta variant with the value of -10.76 kcal/mol (L452R) and -10.38 kcal/mol (T487K) by mediating a consistent hydrogen bond. Y453 and L455 showed nothing or very less contribution to the binding energy for all the three systems. Similarly, Q493, G496, Q498, and Y505 that are mutated to Q493R, G496S, Q498R, and Y505H in the Omicron variant showed the highest contribution to the binding of the Omicron RBD-ACE2 complex with the value of -15.22 kcal/mol, -7.79 kcal/mol, -16.42 kcal/mol, and -12.14 kcal/mol, respectively, which indicated the impact of these residues for higher infectivity and transmission of the Omicron variant.

## Conclusion

Since the emergence of SARS-CoV-2, multiple variants of concern have been reported with increased transmission and viral infectivity. In this study, we investigated the atomistic structural and energetic framework of binding interactions between RBD of SARS-CoV-2 WT, Delta, and Omicron variants with its host cellular receptor ACE2. Accordingly, we simulated the WT, Delta, and Omicron variants in the complex with the ACE2 receptor and evaluated the variations in correspondingly binding energy. The results indicated that the heavily mutated Omicron variant showed the highest binding affinity followed by Delta and WT. The results provide a clear knowledge of the essential molecular determinants in RBD-ACE2 recognition and identified L452R and T478K on Delta RBD while Q493R, G496S, Q498R, and Y505H on Omicron RBD as crucial residues, contributing to the complex stability and rapid spread of the multiple variants. This study paves the way for structure-based drug design of antiviral agents against the spreading multiple variants of SARS-CoV-2.

## Data Availability

The original contributions presented in the study are included in the article/Supplementary Material. Further inquiries can be directed to the corresponding authors.
